# Nano-metals forming bacteria in Egypt. II. Efficacy towards biomolecules, ultrastructure, growth parameters, and eco-friendly therapeutic of soft rot/blackleg genera

**DOI:** 10.1186/s12934-023-02101-6

**Published:** 2023-05-17

**Authors:** Alia A. Shoeib, Nader A. Ashmawy, Ayman Kamal, Sahar Abd El Fatah Zaki

**Affiliations:** 1 Plant Pathology Department, Faculty of Agriculture, Alexandria University, Alexandria, Egypt; 2 Environmental Biotechnology Department, Genetic Engineering and Biotechnology Research Institute, City of Scientific Research and Technological Applications, New Borg El-Arab City, Alexandria, 21934 Egypt

**Keywords:** Nano-Metals, Biomolecules, *Dickeya solani*, Protein, Carbohydrate

## Abstract

The nanoparticles (NPs) formed by *Enterococcus thailandicus, Pseudomonas putida, Marinobacter hydrocarbonoclasticus*, and *P. geniculate* were tested against soft rot/blackleg genera. The effects of NPs recorded on bacterial DNA, proteins, and carbohydrates concentration of *Pectobacterium carotovorum subsp. carotovorum, Enterobacter cloacae* (soft rot), and *Dickeya solani* (soft rot/blackleg). Treated cells showed degradation in isolated DNA, decreased proteins and carbohydrates concentration compared with untreated cells. Using Scanning Electron Microscope (SEM), the treated cells showed collapsed and small pits in the cell wall. Using Transmission Electron Microscope (TEM), internal changes showed penetration of NPs inside the tested bacterial cells, the appearance of periplasmic space, formation of vacuoles, and condensation of cytoplasm. Disease severity *ex vivo* of potato tuber infected with tested genera demonstrated that NPs treatment didn’t show any rotted tissue compared with untreated. The ability to uptake and accumulate FeNPs from the soil in potato (*Solanum tuberosum*) seedlings; Inductively Coupled Plasma-Optical Emission Spectroscopy (ICP-OES) was used. It recorded an increase in iron content of treated potato (*Solanum tuberosum*) seedlings with NPs, compared with untreated. FeNPs can be used to control soft rot/blackleg diseases, instead of copper pesticides. It could be a new, approach for disease management and increase the plant’s nutritional value.

**Supplementary Information:**

The online version contains supplementary material available at 10.1186/s12934-023-02101-6.

## Introduction

The market for potatoes is projected to record a compound annual growth rate (CAGR) of 3.5% during the forecast period 2022–2027. The COVID-19 pandemic has driven the demand for fresh potatoes worldwide in markets and as people stocked up on inexpensive food. The lockdown also increased the request for new potatoes in developing countries [1]. Potatoes are one of the most important crops worldwide, with a global production of 359,071 thousand tons. Potatoes ranked as the world’s sixth most important food crop in production, after sugar cane, maize, wheat, rice, and oil palm fruit. Production of potatoes in Egypt is 5, 216 thousand tons out of 88 799 thousand tons of the total production of primary crop’s main commodities, 2020 [2]. The main challenge will be to produce more potatoes with advanced quality and quantity; at the same time, potatoes are vulnerable to a wide range of pathogenic organisms, all of which can cause severe quality and yield losses. As a result, potato production is highly reliant on pesticide use, and it harms the sustainability of the crop [3]. Subsequently, the European potato production has been reduced by half in the last 60 years, from 221.8 million metric tons in 1961 to 107.3 million metric tons in 2019 [4].

Plant pathogenic bacteria cause different symptoms on different plant organs, e.g. galls, overgrowths, wilts, leaf spots, specks, blights, soft rots, scabs, and cankers [5]. Some produce toxins and inject special types of proteins that lead to host-cell death or enzymes that break down key structural components of plant cells and their walls [6]. Most devastating bacterial plant pathogens species were isolated in Egypt which belong to *Pseudomonas syringae* pathovars [7–9], *Ralstonia solanacearum* [10, 11], *Agrobacterium tumefaciens* [11–13], *Xanthomonas* [14, 15], and *Erwinia amylovora* [16–20]. Egypt is one out of Fifty-six countries that were high in the risk ranking model in their invasion by *Xylella fastidiosa* [21].

Bacterial soft rot soft rots commonly affect vegetables and fruits. It can occur on crops in the field and on the market. Harvesting, handling, and freezing injuries encourage the development of soft rot bacteria in plant tissue [22]. Pectinase, polygalacturonase, and cellulase are enzymes; that play a role in bacterial cell walls degrading, which was excreted by other of bacteria. Decomposition of the cell wall caused by degrading enzymes results in soft rot symptoms. Potatoes crops are exposed to the causative agent of rot symptom, which represents one of the severe diseases in Egypt and around the world. These opportunistic bacterial plant pathogens belong to *Pectobacterium carotovorum* subsp. *carotovorum* and *Enterobacter cloacae* [23–26].

The blackleg disease is responsible for the rotting and wilting of stems on growing potato plants. *Dickeya solani* is a complex disease in Egypt that causes bacterial soft rot/blackleg in potato crops [27]. The symptoms of *D. solani* are often indistinguishable from those caused by *Pectobacterium atrosepticum*, *D. solani* is more virulent as causing disease at lower levels of inoculum, as well as spreading through the plant more effectively [28]. In warm climates, *Dickeya* sp. has also been reported causing blackleg and soft rot of potato [29, 30].

There are many ways to control bacterial plant diseases as using antibiotics. The natural development of bacterial resistance makes the antibiotic ineffective in disease management [31]. Pesticides are caused a hazardous effect on the environment, animals, and human health [32]. The reduction of macro materials into Nano-scale particles (1-100 nm) gives birth to new characteristics of the material that behave differently. Nanomaterials can potentially use in crop protection, especially in plant disease management [33].

Nanomaterials used in plant disease management are a novel approach. It may prove very effective in the future with the progress of the application aspect of agro-nanotechnology [34]. The biological method of nanoparticle (NPs) synthesis is a relatively simple, cheap, and environmentally friendly green chemistry method than the conventional chemical and physical methods [35, 36]. Metallic NPs have demonstrated a broad antibacterial spectrum against both G^**+** ve^, and G^**-**ve^ bacteria due to ultra-small size, high reactivity, large surface area, and different procedure to affect bacterial bioavailability [37].

The objectives of this study applied metallic NPs from eco-friendly bacterial isolates collected from the Egyptian ecosystem on soft rot/blackleg bacteria *ex vivo*. The effect of metal NPs on the content of DNA, carbohydrates, and proteins of the bacterial cell was recorded. The ultrastructure of the interaction of metals NPs with bacterial cells and the presence of metals FeNPs inside the plant tissues were studied. The uptake FeNPs by the potato plant was detected inside the plant tissues.

## Results

### Collection of Nano-metals forming bacteria and soft rot/blackleg genera

Nano-metals forming bacteria *E. thailandicus, P. putida, M. hydrocarbonoclasticus*, and *P. geniculata* for Copper (Cu), Iron (Fe), Cobalt (Co), and Zinc (Zn) nanoparticles (NPs) production sequentially as reported in our previous study Part I [38], were tested for advanced studies on soft rot (*Pectobacterium carotovorum* subsp. *carotovorum* and *Enterobacter cloacae*) and blackleg (*Dickeya solani*) genera, as follow:

### Effect of metals NPs on biomolecules of soft rot/blackleg genera

#### Effect on bacterial DNA

The total DNA, which was isolated from untreated and treated bacterial cells with metallic NPs, was measured. The total bacterial DNA revealed a different amount due to treatment with metal NPs. Single DNA band has shown from untreated bacterial cells **(D)**; and various effects in obtained DNA from treated cells (**S 1**). FeNPs treatment showed total DNA degradation in the case of *P. c.* subsp. *carotovorum* (**S 1 A**), and *E. cloacae* (**S 1 C**), where CoNPs showed whole DNA degradation in the case of *P. c.* subsp. *carotovorum* (**S 1 A**) and *D. solani* (**S 1B**). Also, there was a fragmentation effect of CuNPs and ZnNPs for all tested isolates.

#### Effect on total carbohydrate and proteins

**Fig. 1:**
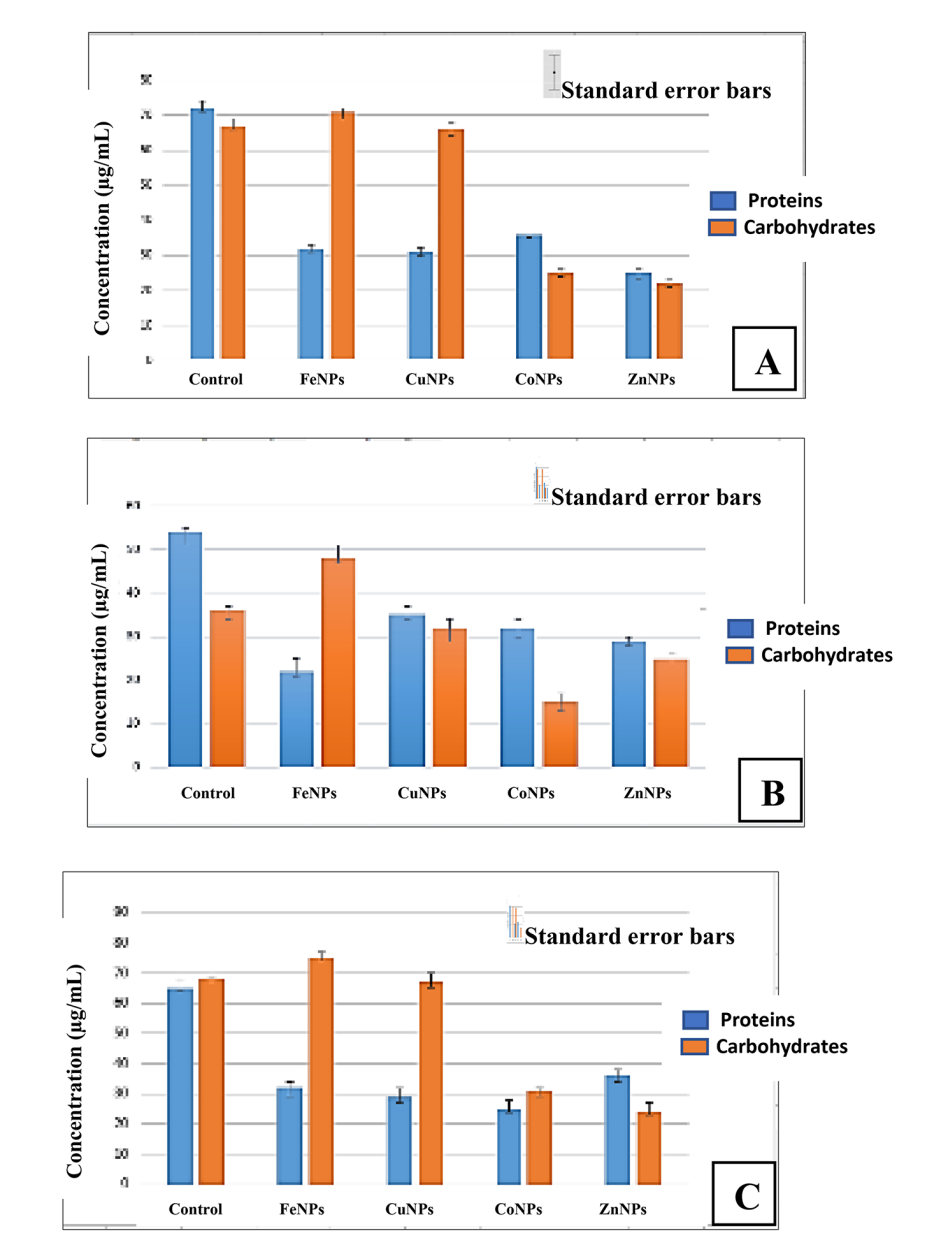
Effect of FeNPs, CuNPs, CoNPs, and ZnNPs on proteins and carbohydrate content of *Pectobacterium carotovorum* subsp. *carotovorum* (A), *Dickeya solani* (B) and *Enterobacter cloacae* (C)

Effects of metal NPs on the metabolic activity of bacterial cells, proteins, and sugars were analyzed. Interaction of metal NPs on total cellular proteins and carbohydrates of phytopathogenic bacteria were in Fig. 1, which illustrated the effect of FeNPs, CuNPs, CoNPs, and ZnNPs on *P. c.* subsp. *carotovorum* (Fig. 1A), *D. solani* (Fig. 1B), and *E. cloacae* (Fig. 1C) compared with untreated bacterial cells. Tested metal NPs showed a significant effect at p ≤ 0.05 on carbohydrate and protein degradations. ZnNPs exhibited a high protein degradation level, followed by CuNPs and FeNPs. CoNPs appeared to reduce significant effect at p ≤ 0.05 on protein degradations. In the case of carbohydrate degradation, ZnNPs and CoNPs were more effective, when compared to the rest of the tested NPs. The effect of metal NPs on total carbohydrate, proteins, and bacterial DNA was tested and performed in three replicates.

#### Ultrastructure effects of metals NPs on bacterial soft rot/blackleg genera

**Fig. 2:**
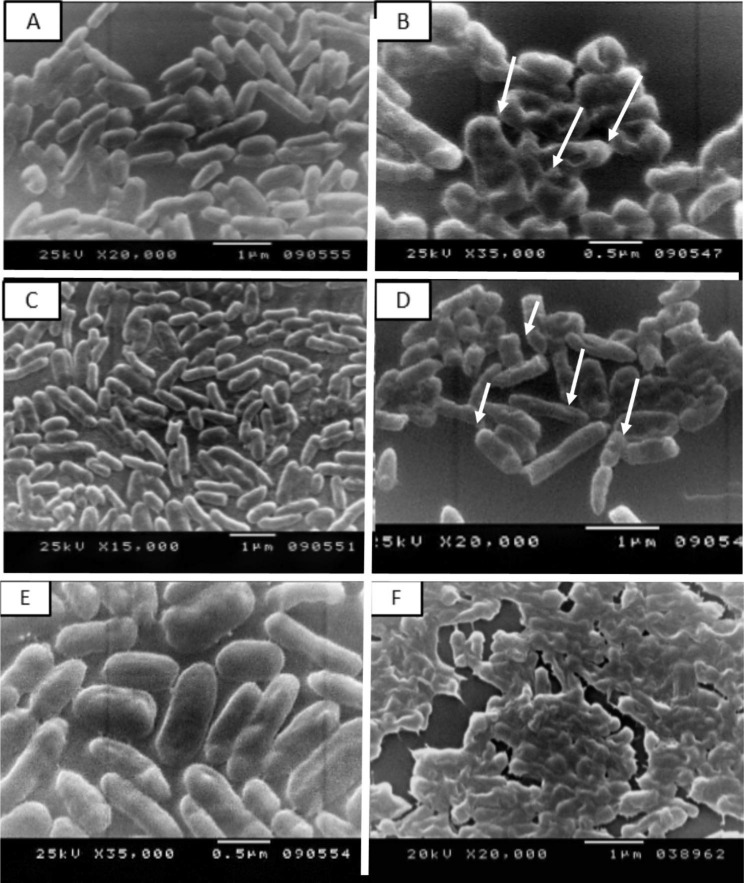
Scanning electron microscope analysis of phytopathogenic bacteria treated with metals NPs. untreated *Pectobacterium carotovorum* subsp. *carotovorum* (A), treated with FeNPs (B), untreated *Dickeya solani* (C), treated with CuNPs (D) and untreated *Enterobacter cloacae* (E), treated CoNPs (F)

**Fig. 3:**
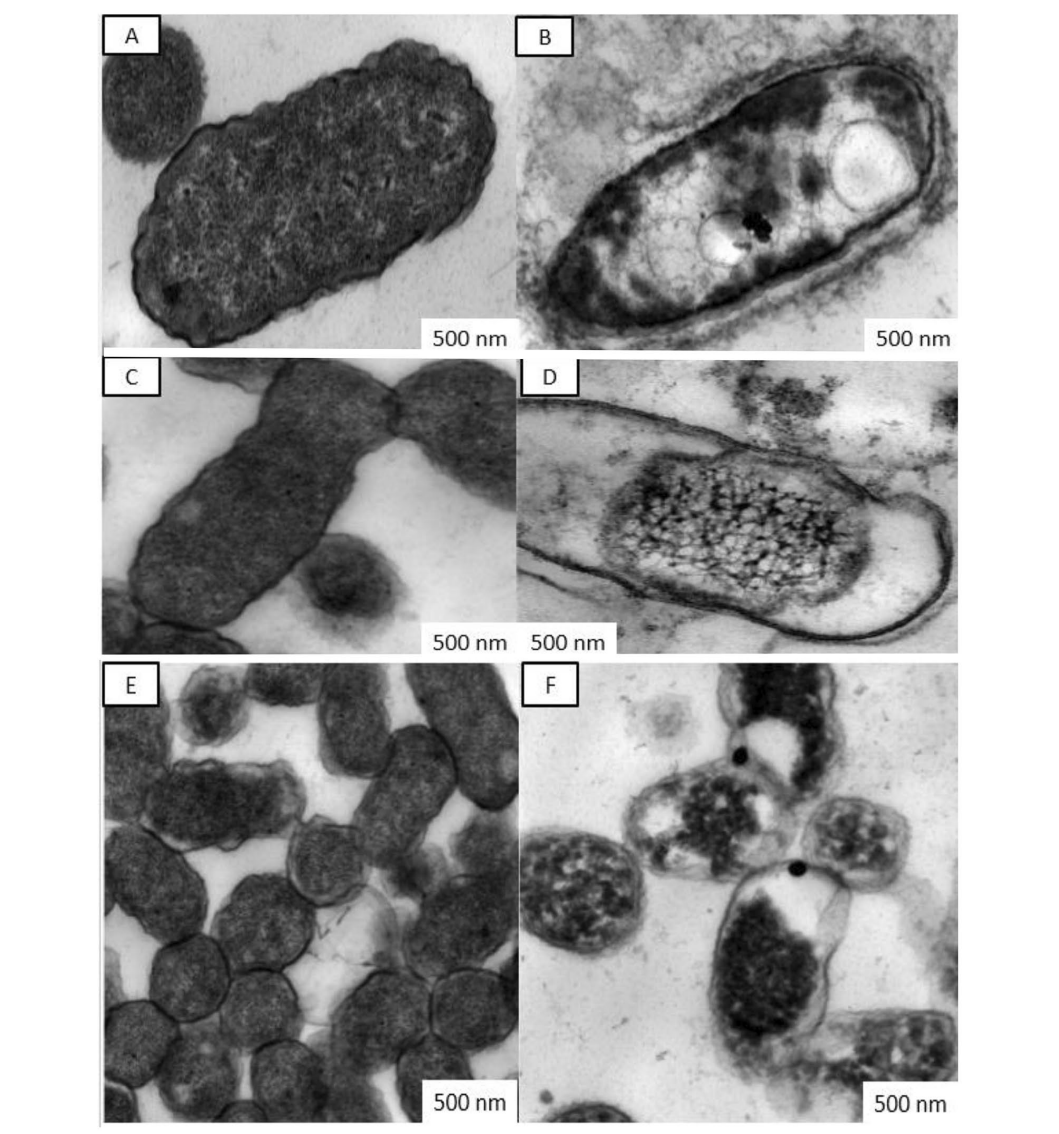
Transmission Electron Microscope analysis of phytopathogenic bacteria treated and untreated with metals NPs. untreated *Pectobacterium carotovorum* subsp. *carotovorum* (A), treated with FeNPs (B*)*, untreated *Dickeya solani* (C), treated with CuNPs (D) and untreated *Enterobacter cloacae* (E), treated with CoNPs (F). NP: NPs, C.W: cell wall, P.S: periplasmic space, P.M: plasma membrane, V: vacuole

Scanning Electron Microscope (SEM) and Transmission Electron Microscope (TEM) were used to examine the morphological and internal changes in bacterial cells treated with metals NPs and as shown in Figs. 2&3.

### SEM observation

The morphological changes of metallic NPs on phytopathogenic bacteria in comparison to untreated cells were achieved using SEM, pictured in Fig. 2. Untreated cells showed smooth, healthy, and damage-free with retained spherical shape cells observed in Fig. 2A, C, E. The treated *P. c.* subsp. *carotovorum* with FeNPs (Fig. 2B) and *D. solani* treated with CuNPs (Fig. 2D) showed a change in the size of the cells, and the big pits in cells in the case of *E. cloacae* treated with CoNPs indicated total lysis and deformation for cells and lost his rod shape as shown in Fig. 2F.

### TEM observation

The internal morphology of treated bacteria shown in Fig. 3 and untreated cells (Fig. 3A, C, E) showed healthy and normal cells in rod- shape with high cytoplasmic density and natural contents. Also, cell walls and plasma membrane were no noticeable changes in morphological structure. Treated cells of *P. c.* subsp. *carotovorum* with FeNPs (Fig. 3B) showed variation in cytoplasmic density compared with untreated cells and noticed the two large vacuoles in the center.

*D. solani* treated with CuNPs in (Fig. 3D) exhibited condensation of cytoplasm in the center of the cell trapped. Additionally, the formation of spacious periplasmic space and plasma membranes were separated from the cell wall and collapsed. E. cloacae treated with CoNPs (Fig. 3F) showed condensation cytoplasm, formation of cytoplasmic space and degradation in the cell wall and swelling in some cells were most probably due to a change in cell permeability. All treated bacterial cells showed penetration and perception of NPs in cells, in addition, to partial lysis of bacterial cell walls of treated genera.

## Application of metals NPs

### Ex vivo effect of metallic NPs on disease severity

**Fig. 4:**
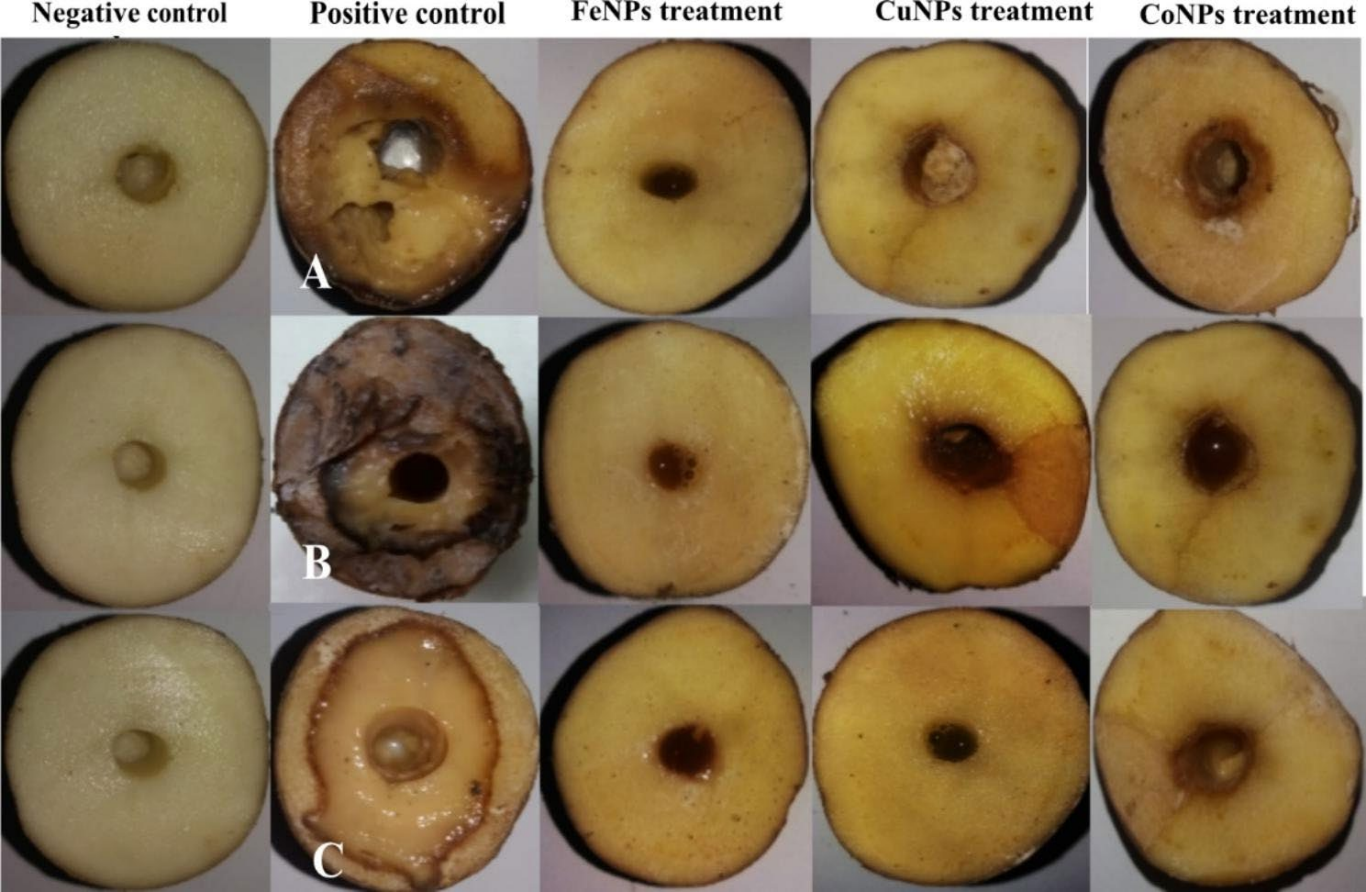
Ex vivo effect of metallic NPs on disease severity of potato tubers ‘Lady Balfour cv.‘ inoculated with *Pectobacterium carotovorum* subsp. *carotovorum, Dickeya solani*, *and Enterobacter cloacae*. Negative control (sterilized water) and positive control (inoculated with pathogenic bacteria) and metallic NPs treatment

Presented data in Table 1; Fig. 4 showed the effect of Fe, Cu, and CoNPs on the disease severity of potato soft rot/blackleg bacteria. Table 1 showed the highest disease severity was in infected potato tuber with *P. c.* subsp. *carotovorum* (47.12), followed by *D. solani* (37.12), and the final rank of *E. cloacae* (32.12). On the other hand, potato tuber treated with FeNPs, CuNPs, and CoNPs showed no soft rot tissues and 0% disease severity. It is worth mentioning color differences around the holes in the treated tuber slices were due to the color of NPs solution (Fig. 4).

**Table 1:** Ex vivo effect of metallic NPs on disease severity of potato tubers ‘Lady Balfour cv.‘ inoculated with *Pectobacterium carotovorum* subsp. *carotovorum, Dickeya solani*, *and Enterobacter cloacae*

NPS Isolate	Positive control	Fe -NPS	Cu-NPS	Co-NPS
*P. carotovorum* subsp. *carotovorum*	47.12	0.0	0.0	0.0
*D. solani*	37.12	0.0	0.0	0.0
*En. cloacae*	32.12	0.0	0.0	0.0

### In vivo effect of FeNPs on growth parameter of infected potato plants

**Fig. 5:**
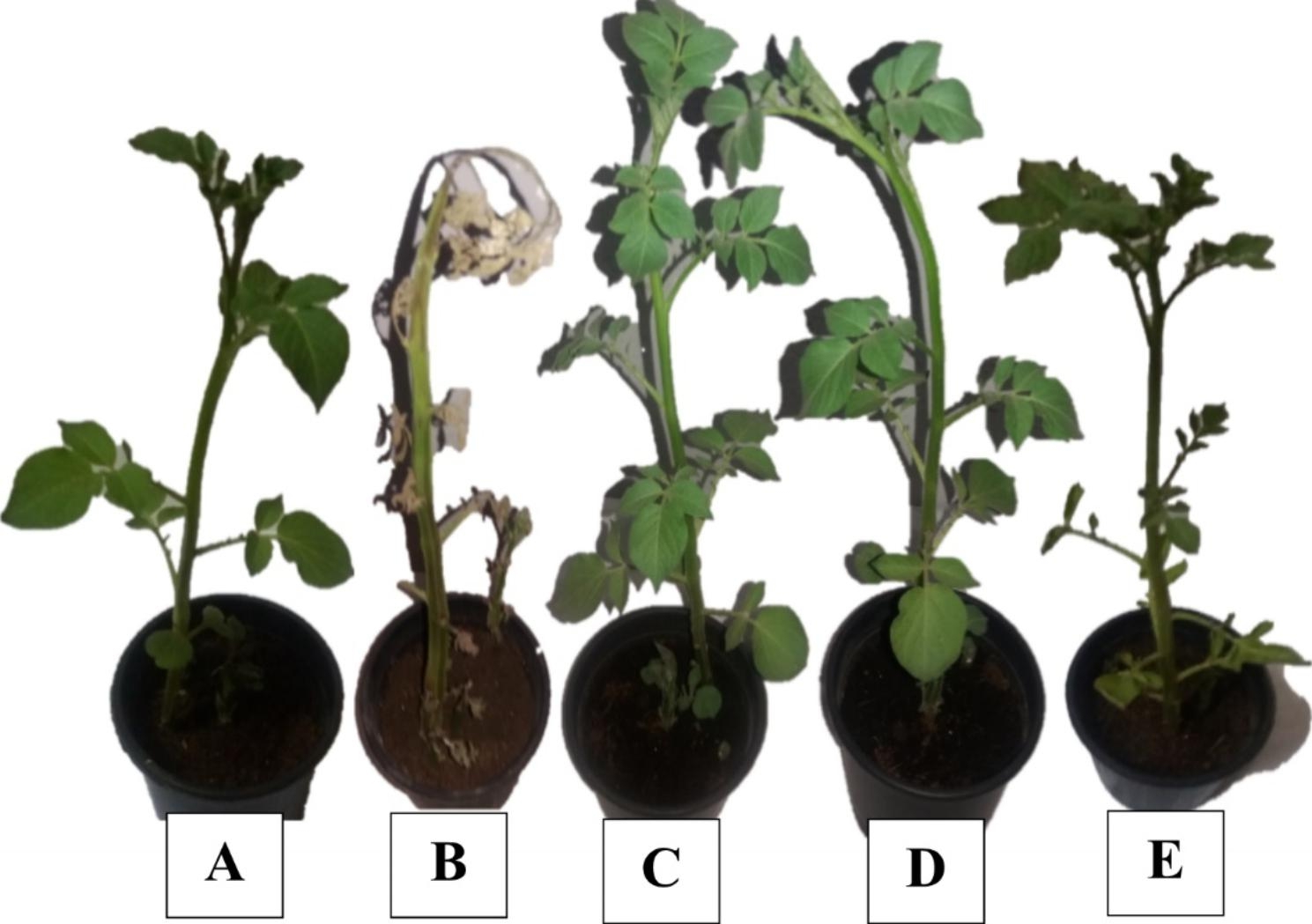
Effect of NPs treatment on shoot system of potato plant ‘Lady Balfour cv.‘ inoculated with *Dickeya Solani.* A: Negative control, B: Positive control, C: FeNPs control, D: FeNPs treatment, E: Copper pesticide treatment

**Fig. 6:**
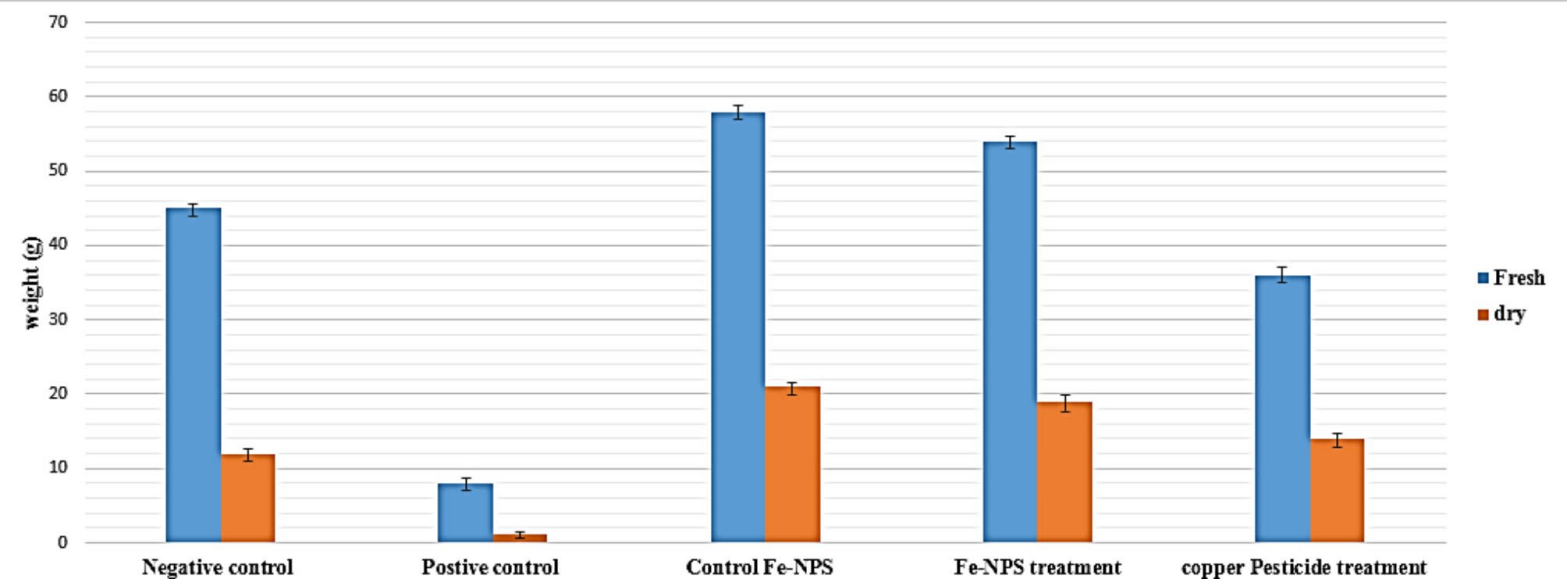
In vivo effect of FeNPs on growth parameter fresh and dry weight of potato seedlings ‘Lady Balfour cv.‘ infected with *Dickeya solani*

The effect of FeNPs on the growth parameters of infected potato plants was listed (Table 2, and Figs. 5 and 6), compared with those treated with copper pesticide and healthy plants without any treatment. The NPs treatment showed a highly significant increase in fresh and dry weight of potato seedlings ‘Lady Balfour cv., 412.14%, and 161.93%, respectively, compared with infected plants (389.08%, and 139.29%, respectively) as a moderate significant increase. Subsequently, the copper pesticide treatment showed a lower significant increase in fresh weight (217.45%) and dry (78.58%) weight of infected potato seedlings.

**Table 2:** In vivo effect of FeNPs treatment on growth parameter (fresh and dry weight) of potato seedlings ‘Lady Balfour cv.‘ infected with *Dickeya solani*

Growth parameter Treatments	Fresh weight (g)	Increase %	Dry weight (g)	Increase %
Healthy plant (Negative control)	45.38^c^*	387.51	12.07^d^	47.73
Infected plant by *Dickeya solani* (Positive control 1)	11.36^e^	-	8.17^e^	-
FeNPs treatment (Positive control 2)	58.18^a^	412.14	21.40^a^	161.93
*D. solani* + FeNPs treatment	55.56^b^	389.08	19.55^b^	139.29
*D. solani* + Copper pesticide treatment	36.04^d^	217.45	14.59^c^	78.58

*Means with Common letters are not significant (i.e., Means with Different letters are significant), statistically significant at p ≤ 0.05

### The ability of potato plant to uptake FeNPs

**Fig. 7:**
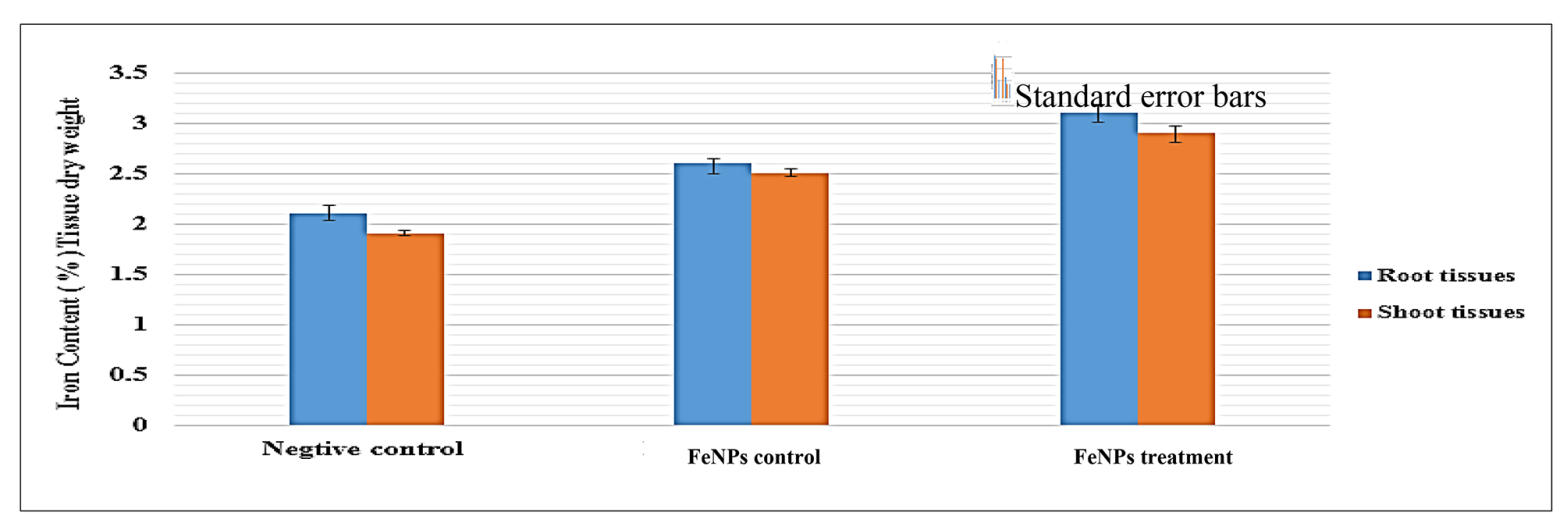
Iron uptake (%) by using Inductively Coupled Plasma-Optical Emission Spectroscopy (ICP-OES) in potato seedlings ‘Lady Balfour cv.‘ as a negative control, infected seedlings with *Dickeya solani* + treated with FeNPs, and treated seedlings with FeNPs as a positive control

Inductively Coupled Plasma-Optical Emission Spectroscopy (ICP-OES) was used to measure the uptake of FeNPs by potato seedlings. The uptake FeNPs was recorded by using treated roots and shoots of seedlings with FePNs, and infected seedlings with *D. solani* + FeNPs treatment compared with untreated ones (healthy seedlings). The iron content (%) in root and shoot tissues was assayed in either infected plants with *D. solani* (treatment) or treated plants with FeNPs (Positive control) in comparison to the healthy plants (Negative control). The results revealed an increasingly significant iron content of treated seedlings compared with either untreated or *D. solani* + FeNPs treatment (Fig. 7).

## Discussion

Nanoparticles (NPs) have different properties compared to metallic or micro-particles. The effects of Fe, Cu, and CoNPs on DNA degradation, in treated *P. carotovorum* subsp. *carotovorum*, *D. solani*, and *E. cloacae* were examined. Results of that study revealed that metal NPs had a damaging effect on genomic DNA, leading to degradation and fragmentation. Jose et al., [39] proposed a mechanism of DNA damage through the generation of singlet oxygen as reported in the case of CuNPs. Wang et al., [40] described that the antibacterial activity of Fe, Fe oxide, Cu, Zn, and CoNPs has different mechanisms. It affects cellular leakage, reactive oxygen species production (ROS), cell membrane damage, inhibits the formation of bacterial biofilms, inhibits the synthesis of bacterial proteins and DNA, binding and damage to cellular DNA and DNA repair, sulfur-related proteins, and metabolic genes. Nejdl et al., [41] reported that platinum nanoparticles (PtNPs) platinum nanoparticles (PtNPs)inhibited DNA replication and interacted with the bacterial DNA of *Salmonella enteritidis*. He noted that DNA secondary structures due to DNA degradation could block transcription and replication with subsequent apoptosis. Rafi et al., [42] reported that the antibacterial activity of iron oxide NPs (IONPs) is via oxidative stress generated by ROS, it’s resulting in the damage of the proteins and DNA in the bacteria.

Effect of metal NPs on biomolecules contents of tested bacteria demonstrated ZnNPs have a high level of protein break down followed by Co and FeNPs. Zn and CoNPs were more effective on carbohydrate degradation. FeNPs treatment showed an increase in carbohydrate content of *P. c.* subsp. *carotovorum* and *E. cloacae*. The initial hypothesis was that the increase in carbohydrates was due to a raise in capsular carbohydrates [43]. Result with *Escherichia coli* B23 cell treatment with streptomycin and kanamycin [44]. Yuan et al., [45] recorded that the amounts of protein released in the suspension of the AgNP treated G^− ve^*P. aeruginosa* and *E. coli* a significantly higher compared with G^+ ve^*S. aureus* cells. The antibacterial activity of copper oxide-based NPs attributed to the following: the generation of ROS, protein oxidation, lipid peroxidation, destruction of the cell membrane, and DNA degradation in bacteria cells [46]. Li et al., [47] reported that *E. coli* cells treated with AgNPs showed leakage of reducing sugars of bacterial dry weight due to the higher concentration of AgNPs. It has been antibacterial effects through influencing membrane permeability, and inducing the leakage of reducing sugars, which it leading to bacterial cell death [45, 48].

TEM and SEM studies on the ultrastructure effect of metal NPs on *P. c.* subsp. *carotovorum*, *D. solani*, and *E. cloacae* treated with Fe, Cu, and CoNPs have been tested. Results concluded that NPs could attack the bacterial cell-making pits, deformation, lysis, and cellular leakage, in addition to making some vacuoles and periplasmic space. Metal NPs had a lethal biocide effect on G-ve phytopathogenic bacteria. Kamal et al.,[36] and Soo-Hwan et al., [49], observed the disruptive effects of NPs. In addition, AgNPs lead to the formation of pits in the cell walls of the bacteria, which could enter into the periplasm through these pits and destroy the cell membrane. They reported that NPs anchor into the cell membrane and enter the cells, leading to osmotic collapse and subsequent release of intracellular materials. Several mechanisms of NPs, once deposited on the microbial surface, including cell wall perforation and morphological changes as irregular-shaped pits [50]. NPs cause cell membrane detaching from the cell wall, destabilization, and pits induce, this leads to a rapid increase in cell permeability and intra-component leakage [51]. Once NPs penetrate cell barriers into the entire cell, NPs interact with phosphorus-containing compounds as DNA, which causes losing their replication ability and inhibits DNA unwinding [52]. The damaged cells were examined using TEM; which revealed that the cell wall separated from the internal cellular components and electron-dense aggregation of compounds was surrounding the lysed cell [53]. The thickness of the peptidoglycan layer in G^+ ve^ bacteria has an essential *role* in protecting the cell from NPs impregnation [54, 55].

Present data on the effect of NPs on disease severity of potato tuber ex vivo infected with *P. c.* subsp. *carotovorum*, *D. solani*, and *E. cloacae* concluded that Fe, Cu, and CoNPs decreased disease severity by 100% due to their antibacterial activity. The impact of Ag and SeNPs decreasing disease severity of early blight disease in potatoes caused by *Alternaria solani* and improved plant parameters included physiological parameters and yield [56]. A wide range of nanotechnology applications emerged into the agri-food-sector, including Nano-sensors, tracking devices, targeted delivery of required components, food safety, new product developments, precision processing, packaging, and others [57–59].

Effect of FeNPs on growth parameters of potato seedlings treated with FeNPs had positive effects on controlling soft rot/blackleg disease caused by *D. solani*. Additionally, positive impact on growth weight parameters as an increase in fresh and dry weight. Iron is an essential micronutrient for almost all living organisms because it plays a critical role in DNA synthesis, respiration, photosynthesis, a prosthetic group constituent of many enzymes, and chlorophyll synthesis, and it’s essential for the maintenance of chloroplast structure and function [60]. The Iron oxide NPs (Fe_3_O_4_ NP) at lower concentrations have a beneficiary impact on the plant and improve germination [61, 62].

The uptake NPs in plant tissue was evaluated using ICP-OES, which it’s demonstrated an increase in iron content in healthy plants treated with FeNPs, followed by infected plants with *D. solani* compared with negative control treated with pure distilled water. It might be referred to as infected plants which uptake a high amount of Fe NPs. Nwugo et al. [63] pointed out that stressed plant tissues could accumulate more nutrients/unit mass than unstressed tissues. Extension potato plant infected with *Candidatus* Liberibacter solanacearum induced nutrient accumulation was detected for micronutrients, especially iron in leaf and root tissues. Plant cell walls are composed of cellulose which permits the entry of small particles and restricts the larger ones; therefore, smaller NPs can enter through this layer. The size exclusion limit for the plant cell wall is between 5 and 20 nm [64]. Etxeberria et al. [65] informed that NPs might move through endocytosis and further through the symplastic transport; they might travel to different plant tissues. Wang et al. [66] indicated that size, magnitude, and zeta potentials are keys in determining the transport of NPs inside the plant.

Depending on the results obtained from our findings in the previous study [38], it proved the ability of FeNPs to act as antibacterial agents of *D. solani*. Thus FeNPs were chosen in this study to test the uptake in plant tissue. As well, electro-microscopic images showed lysis in the treated cells with FeNPs. Based on the above, we suggest that the degradation of pathogenic bacterial cells has an effect on adding more nutrients to plants plus treating them with FeNPs. Therefore, the treatment with FeNPs of infected seedlings with D. solani; leads to growth parameters nearly than the FeNPs treatment only. Islam et al. [67] reported that cellular disruption or cell lysis is a method in which the outer boundary or cell membrane is broken down or destroyed to release inter-cellular materials such as DNA, RNA, protein, or organelles from a cell.

## Conclusion and recommendations

A biological synthesis of NPs from bacterial cells is eco-friendly, fast, and inexpensive, as well as the high toxicity of Fe, Cu, Co, and ZnNPs against phytopathogenic bacteria.

FeNPs are one of the best nanoparticles that give effectiveness to pathogenic bacteria, as a vital micronutrient for plants and beneficial bacteria, which plays a critical role in metabolic processes. FeNPs are best selected to control soft rot/blackleg diseases in potato plants ex vivo. Additionally, FeNPs promote the growth of potato seedlings better than copper pesticides.

Generally, Nanomaterials will increase the efficacy of pesticides and antibiotics, allowing a decrease in the doses used. Therefore, we recommend adding it to the irrigation water to nutrient the plant and enhance its resistance to diseases in general and to resist soft rot diseases. Utilization of metal NPs in the bactericide industry, more in vivo experimental tests for the toxicity and safety concentrations of metal NPs in animals and human cells are required.

## Materials and methods

### Source of Nano-metals forming bacteria

Bacterial isolates from harsh conditions locals (industrial wastewater, seawater, wastewater, and lake water); were collected from Alexandria, Hurghada, and Damietta Governorates in Egypt. These bacteria were isolated in a previous study by the authors and published by Zaki et al. [38]. Four selected isolates identified with accession No. as *E. thailandicus*, *P. putida*, *M. hydrocarbonoclasticus*, and *P. geniculate* for Copper (Cu), Iron (Fe), Cobalt (Co) and Zinc (Zn) nanoparticles (NPs) production sequentially (Table 3).

**Table 3:** Identification of Nano-metals forming bacteria isolates and accession number

Genus	Accession No.
*Enterococcus thailandicus*	MG831199^38^
*Marinobacter hydrocarbonoclasticus*	MG83323^38^
*Pseudomonas putida*	MG833008^38^
*Pseudomonas geniculata*	MG83172^38^

The efficacy of metal NPs against the three molecular identified phytopathogenic bacteria; was determined. The bacteria that cause soft rot disease (*P. c.* subsp. *carotovorum*, *Enterobacter cloacae*), and blackleg disease (*Dickeya solani*), were kindly obtained by Shams et al. [25] (Table 4).

**Table 4:** Soft rot and blackleg bacterial isolates used in this study

Bacterial isolates	Accession No.
*Pectobacterium carotovorum* subsp. *carotovorum*	LN811442^25^
*Dickeya solani*	LT592259^25^
*Enterobacter cloacae*	LT592256^25^

### Production of NPs

A pick of the single colony of separately isolate was inoculated in 20 ml Luria-Bertani (LB) [68] broth, which was incubated at 30 °C in a shaking incubator at 150 rpm for 24 h. The broth culture adjusted to being 0.5 McFarland standard. Ten mL of each culture was inoculated in 100 mL LB broth in a 500 mL Erlenmeyer flask supplemented with 3.5 mM of either Fe (NO_3_) 3·9H2O, Cu (NO_3_)_2_. 3H_2_O, Co (NO_3_)_2_.6H_2_O or Zn (NO_3_)_2_.6H2O, then incubated in a shaking incubator at 150 rpm at 30 °C until the color of cultures became dark brown within 4–7 days according to Zaki et al. [35].

### Extraction of metals NPs

Metallic NPs are formed inside various bacterial cell origins, such as the cell wall, between the cytoplasm and plasma membrane, and floating in the cytoplasm. Consequently, the cells disrupted to release metallic NPs, for the analysis and a different applications. The ultrasonic disruption method, has been used as a physical technique. The bacterial cell culture (50mL) containing NPs, was centrifuged at 1006 xg in Hermle Universal Centrifuge Z 306 for 30 Min. The cells pellet was washed with sterile water and dried in an oven for 48 h on 60°*C*, then re-suspended in 50 mL of sterilized Milli Q water. The sonication was achieved for 30 Min, on/off cycle, for 59 s on Vibra-Cell™, Ultrasonic Liquid Processors Sonics and materials VC 505/VC 750. The sample sonication was centrifuged for 30 s at 1006 xg to separate the cell debris. The supernatant that contained a suspension of NPs; was lyophilized using a freeze dryer Lyophilizer [36].

### Effect of metals NPs on biomolecules

Bacterial isolates with ca.1 × 106 CFU/mL were treated with either Fe, Cu, Co, or ZnNPs, and incubated overnight at 30 °C. The control group in an experiment is the group in which the cultures are free from metal NPs. The cell lysates preparation methods have been set according to Park et al. [71].

### Effect of metals NPs on bacterial DNA

To study the effect of metallic NPs on bacterial DNA, cells were treated with Fe, Cu, Co, and Zn NPs separately, and incubated overnight at 30 °C. DNA was extracted from treated and untreated cells as control with the AMSHAGE DNA extraction kit. Bacterial DNA content was determined three times and three replicates.

### Effect of metals NPs on total proteins

Protein was determined according to Lowry et al., [74] methods, using bovine serum albumin (BSA) as a standard protein. The solutions A, B, C, and D [A: 2% Na2CO3 in 1%M NaOH; B: 0.5% CuSO_4_ in 1% (w/v) sodium tartrate; C: Mix of 50mL of reagent A with 1mL of reagent B, and D: Folin’s reagent (BOH) diluted with water 1:3] was prepared. A protein sample (0.1) of the cell-free extract was added to 5 mL of solution C, mixed well, and allowed to stand for 10 Min. Half mL of solution D was added with mixing and allowed to stand for another 30 Min to allow the color to develop. The absorbance of the sample was measured at 750 nm in the spectrophotometer T60 UV/VIS. Total proteins were carried out three times and three replicates.

### Effect of metals NPs on total carbohydrate

Total soluble carbohydrates were determined using the anthrone technique [72]. Free cells supernatant (three mL) was transferred; to a clean test tube. Add freshly prepared (six mL) anthrone reagent (2 g anthrone/L of 95% sulphuric acid). These tubes were heated in a boiling water bath for 3 Min and left to cool. The developed color was measured using a spectrophotometer (T60 UV/VIS Spectrophotometer) at 620 nm. The distilled water and reagent as a blank mixture under the same condition were measured. A standard curve was created, using glucose as a standardized carbohydrate from which sugar concentrations were determined [73]. This *experiment* was *done three times*, with three replicates.

### Ultrastructure effect of metals NPs on some bacterial soft rot/blackleg genera

Treated bacterial isolates with metals NPs; were harvested for preparation and examination by transmission and scanning electron microscopy, according to Park et al. [69] and Iwasawa et al. [70]. The isolates, after accumulation and reduction of metallic ions, were collected. Glutaraldehyde (2%) and formaldehyde (4%), in phosphate buffer saline (PBS), at 4 °C overnight were used to fix the cells. The fixed cells were washed the three-time (each for 10 Min) with 0.1 M sodium cacodylate buffer (pH 7.4). The samples were post-fixed with 1% (v/v) osmium tetroxide at 4 °C for 2 h. Then, the post-fixed cells were washed three times (each time for 10 Min) with 0.1 M sodium cacodylate buffer (pH 7.4).

In the ultra-section’s preparation, the dehydrated post-fixed samples in an ascending acetone concentration, from 35 to 95% (each for 10 Min). The samples were dehydrated in acetone 100% three times (each for 15 Min). This sample in Epon 812 was embedded, then polymerized in an oven at 60 °C for 24–48 h. Upon was cut with glass knives, staining using uranyl acetate for 10 Min and lead citrate stain for 10 Min. At the final point, it used TEM (JSM 1400 plus -JEOL) for ultra-sections examination.

The post-fixed samples in a series of graded ethanol series (from 30 to 90%) each for 10 Min; it’s dehydrated in absolute ethanol three times (15 Min each). The dehydrated samples were critical point dried (Samdri PVT-3B Critical Point Dryer) for 30 Min. The dried specimen was coated with gold 90%/10% w/w using a sputter coater (Jeol Fine Coat JFC-1100E). SEM (JEOL 5300 JSM) was used to examine the samples.

## Application of metals NPs

### Ex vivo effect of metals NPs on disease severity

Disease severity was estimated according to Schober and Vermeulen [75], as a percentage of rotted tissue weight according to the change of tuber weight before and after treatment divided on the weight of tuber before treatment as the following formula:

^*^PDS = (W1-W2)/W1 × 100

* Whereas: PDS = percentage of disease severity, W1 = weight of the whole tuber before treatment and W2 = weight of tuber after removal of the rotten tissue.

*P. c. subsp. carotovorum* and *E. cloacae*, causing the soft rot; *D. solani*, causing the soft rot/blackleg; were utilized. The healthy potato (*Solanum tuberosum*) tuber ‘Lady Balfour cv.‘; was brought from fresh market potatoes at Alexandria Governorate. This market is available for the public to purchase, and they do not need permission to obtain the product. All study/experimental protocols involving plant materials were conducted by institutional, national, and international guidelines and legislation. This was done by surfaced-sterilized potato tuber for 10 Min with 1% (v/v) sodium hypochlorite solution, rinsed thoroughly, and allowed to air dry. For each isolate, 3 tubers were cut in half; as well as, a hole was formed in half tuber center, ca. 1 cm in deep, with a sterilized Cork borer (1 cm in diameter). The 250 µL of culture ca.1 × 106 CFU/mL (OD600 = 0.7) bacterial suspensions were prepared; from 24 h; and then placed into the wound [76]. Sterile distilled water was used as a negative control. Potato tubers have been set randomized in plastic trays supplemented with sterilized moist cotton to maintain high humidity and incubated for 48 h at 28 + 2 ° C after inoculation. Rotting tissue was removed from the potato tubers with a sterile spatula [77].

In the case of NPs treatment, potato tubers were submerged with Fe, Cu, and Co NPs (300 µg/mL) separately; before the tubers were treated with a bacterial suspension (1 × 106 CFU/mL). The sterile distilled water has been used, as a negative control. Treated potato tubers were placed in plastic trays randomly with sterilized moist cotton. The data of the experiments, after being incubated for 48 h at 28 + 2 °C were measured.

### In vivo effect of FeNPs on growth parameter of infected potato seedling

FeNPs and soft rot/blackleg bacteria *D. solani* were selected as a model of in vivo experiment to control the bacterial plant diseases in potato plant. Surfaces of the potato tubers were sterilized with 1% sodium hypochlorite for 5 Min, washed with sterile water. The treated tubers planted (one tuber/pot) in 15 cm in external diameter; filled with sterilized peat moss and clay (1:1) [78]. The suspension (0.5 mL of 1 × 106 CFU/mL (OD600 = 0.7)/pot) of *D. solani* injecting into the soil; when potato plants reached 15–20 cm in length.

In case of open-air for dry, one mL of each FeNPs (300 µg/mL)/pot) and index (77% copper hydroxide)/pot [79] were added to the soil separately. Subsequently, after two days, the pots were inoculated with a suspension of bacteria. The plant’s irrigation with sterile distilled water served as a control. Inoculated seedlings were placed directly in a greenhouse at 25 ± 2ºC. The *g*rowth parameters were recorded after 14 days of the bacterial inoculation, as root and shoot fresh and dry weight. Four replicates were applied to measure variation in this experiment.

### The ability of potato plants to uptake and accumulate of NPs

The ability of the potato plants to accumulate NPs; by Inductively Coupled Plasma-Optical Emission Spectroscopy (ICP-OES) was tested according to Banuelos et al. [80]. The hole plant as leaves, roots, stems, and tubers were washed with double distilled water, placed in beakers, then covered with watch glasses, dried for a 12 h in an oven at 110 °C. Later these samples were triturated to be homogenized well.

For sample digestion, approximately 0.50 ± 0.01 g per dry sample added into 50-mL cleaned and air-dried Folin tube; finally, 5 mL concentrated nitric acid was supplementary. These samples placed at room temperature for 2–3 h, then Folin tubes were kept in a heating block at 120–130ºC for 14–16 h. The specimens were let cool for several Min, then one mL; of 30% hydrogen peroxide was added per each specimen. Samples were placed back onto the heating block for 20–30 Min. Water was added to the 50 mL mark and let sit for 30 Min.

To analyze ICP-OES, samples were diluted, and analyses were performed on Agilent ICP-OES 5110 VDV. The ICP-OES system was calibrated by serial dilutions of Fe, with limits of detection (10–1000 µg/mL5a). The emission lines used for the analyses were 238.20 nm, under Argon plasma with the concentric nebulizer.

### Statistical analysis

Data were fed to the computer and analyzed using IBM SPSS software packages version 20.0. **(**Armonk, NY: IBM Corp**)**. The Kolmogorov-Smirnov test was using for verifying the normality of distribution. Quantitative data, using the mean and the standard deviation significance, was judged at the 5% level. The used test was F-test (ANOVA); for normally distributed quantitative variables; to compare between more than two groups and the Post Hoc test (LSD) for pairwise comparisons.

## Electronic supplementary material

Below is the link to the electronic supplementary material.

**Additional file 1:** Effect of CuNPs, FeNPs, CoNPs, and ZnNPs on DNA of *Pectobacterium carotovorum subsp. carotovorum* (A), *Dickeya solani* (B), and *Enterobacter cloacae* (C), DNA from untreated bacterial cells (D), and 100 bp DNA ladder (M).

## Data Availability

The datasets used and/or analyzed during the current study are available from the corresponding author upon reasonable request.
