# Supplementary material for: Nano-metals forming bacteria in Egypt. II. Efficacy towards biomolecules, ultrastructure, growth parameters, and eco-friendly therapeutic of soft rot/blackleg genera
Source: Microb Cell Fact. 2023 May 17;22:101. doi: 10.1186/s12934-023-02101-6 (PMC10189210; doi:10.1186/s12934-023-02101-6)
Supplement: Supplementary file 1 — Additional file 1: Effect of CuNPs, FeNPs, CoNPs, and ZnNPs on DNA of Pectobacterium carotovorum subsp. carotovorum (A), Dickeya solani (B), and Enterobacter cloacae (C), DNA from untreated bacterial cells (D), and 100 bp DNA ladder (M). [file 12934_2023_2101_MOESM1_ESM.docx]

# Nano-Metals Forming Bacteria in Egypt. II. Efficacy towards Biomolecules, Ultrastructure, Growth Parameters, and Eco-Friendly Therapeutic of Soft Rot/Blackleg Genera

**Alia A. Shoeib^1*^, Nader A. Ashmawy^1^, Ayman Kamal^2^ & Sahar Abd El Fatah Zaki^2^**

^1^Plant Pathology Department, Faculty of Agriculture, Alexandria University, Alexandria, Egypt.

^2^Environmental Biotechnology Department, Genetic Engineering and Biotechnology Research Institute, City of Scientific Research and Technological Applications, Alexandria, Egypt.


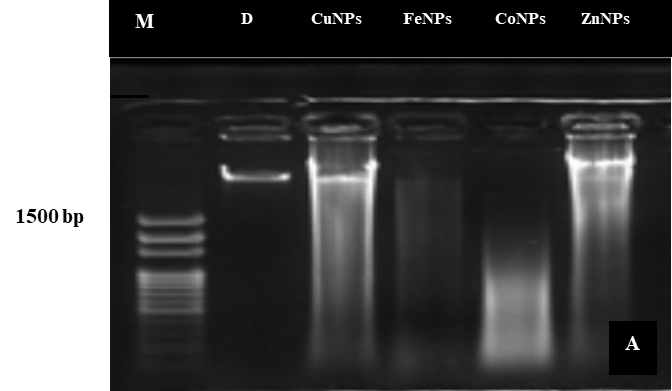


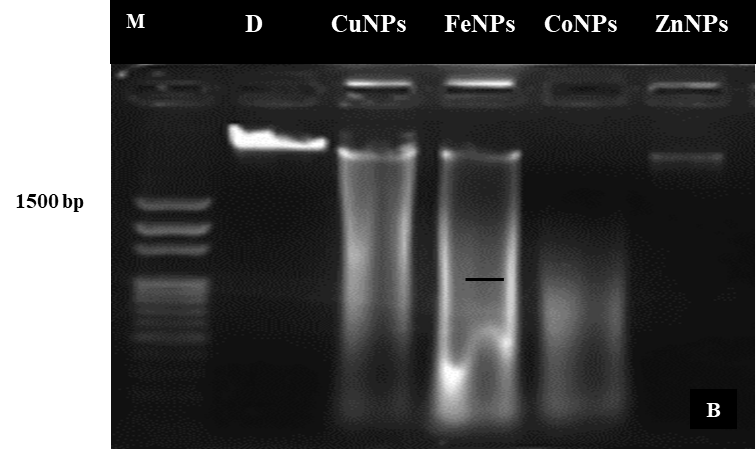


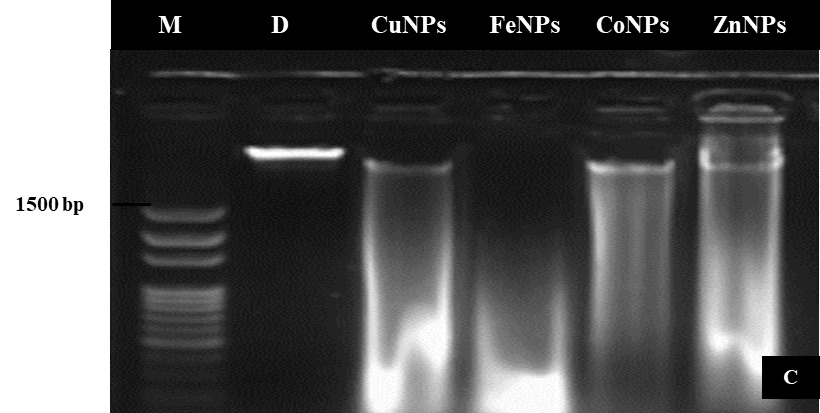


**S 1.** Effect of CuNPs, FeNPs, CoNPs, and ZnNPs on DNA of Pectobacterium carotovorum subsp. carotovorum (A), *Dickeya solani* (B), and *Enterobacter cloacae* (C), DNA from untreated bacterial cells (D), and 100 bp DNA ladder (M).

…………………………………………………..…………………………………………………………………………………
